# Beneficial Effects Induced by a Proprietary Blend of a New Bromelain-Based Polyenzymatic Complex Plus N-Acetylcysteine in Urinary Tract Infections: Results from In Vitro and Ex Vivo Studies

**DOI:** 10.3390/antibiotics13100985

**Published:** 2024-10-18

**Authors:** Lucia Recinella, Morena Pinti, Maria Loreta Libero, Silvia Di Lodovico, Serena Veschi, Anna Piro, Daniele Generali, Alessandra Acquaviva, Nilofar Nilofar, Giustino Orlando, Annalisa Chiavaroli, Claudio Ferrante, Luigi Menghini, Simonetta Cristina Di Simone, Luigi Brunetti, Mara Di Giulio, Sheila Leone

**Affiliations:** 1 Department of Pharmacy, “G. d’Annunzio” University, 66013 Chieti, Italy; lucia.recinella@unich.it (L.R.); morena.pinti@phd.unich.it (M.P.); silvia.dilodovico@unich.it (S.D.L.); veschi@unich.it (S.V.); anna.piro@unich.it (A.P.); alessandra.acquaviva@unich.it (A.A.); nilofar.nilofar@unich.it (N.N.); giustino.orlando@unich.it (G.O.); annalisa.chiavaroli@unich.it (A.C.); claudio.ferrante@unich.it (C.F.); luigi.menghini@unich.it (L.M.); simonetta.disimone@unich.it (S.C.D.S.); luigi.brunetti@unich.it (L.B.); mdigiulio@unich.it (M.D.G.); sheila.leone@unich.it (S.L.); 2 Department of Medical, Surgical and Health Sciences, University of Trieste, 34149 Trieste, Italy; dgenerali@units.it; 3 Department of Advanced Translational Microbiology, Institute for Maternal and Child Health-IRCCS “Burlo Garofolo”, 34137 Trieste, Italy

**Keywords:** bromelain, N-acetylcysteine, adhesion capability, biofilm, oxidative stress, inflammation

## Abstract

**Background/Objectives:** Urinary tract infections (UTIs) are infections that involve the urethra, bladder, and, in much more severe cases, even kidneys. These infections represent one of the most common diseases worldwide. Various pathogens are responsible for this condition, the most common being *Escherichia coli* (*E. coli*). Bromelain is a proteolytic complex obtained from the stem and stalk of *Ananas comosus* (L.) Merr. showing several beneficial activities. In addition to bromelain, N-acetylcysteine (NAC) has also been used. **Methods:** The purpose of this experiment was to evaluate the antibacterial, anti-motility, and anti-biofilm effects of a new polyenzymatic complex (DIF17BRO^®^) in combination with NAC (the Formulation) on various strains of *E. coli* isolated from patients with UTIs. Subsequently, the anti-inflammatory and antioxidant effects of the Formulation were studied in an ex vivo model of cystitis, using bladder samples from mice exposed to *E. coli* lipopolysaccharide (LPS). **Results:** Our results showed that the Formulation significantly affects the capability of bacteria to form biofilm and reduces the bacteria amount in the mature biofilm. Moreover, it combines the interesting properties of NAC and a polyenzyme plant complex based on bromelain in a right dose to affect the *E. coli* adhesion capability. Finally, the Formulation exhibited protective effects, as confirmed by the inhibitory activities on multiple inflammatory and oxidative stress-related pathways on bladder specimens exposed to LPS. **Conclusions:** This blend of active compounds could represent a promising and versatile approach to use to overcome the limitations associated with conventional therapies.

## 1. Introduction

Urinary tract infections (UTIs) are infections involving the urethra, bladder, or, in severe cases, kidneys. They represent one of the most common infection diseases with high morbidity and more healthcare costs in the world [1]. UTIs have a negative impact on the patient’s health decreasing social relationships and quality of life [2,3].

Different microbial pathogens are involved in the pathophysiology of the UTIs, such as *Enterobacteriaceae*, *Pseudomonas*, *Staphylococci*, and Fungi. However, *Escherichia coli* (*E. coli*) is frequently responsible for about 80% of all infections of the lower urinary tract which affect the bladder and is frequently involved in biofilm-related diseases inducing relapses or chronic infections [4,5,6].

Pharmacological treatment of UTIs includes antimicrobial agents, such as trimethoprim and sulfamethoxazole, β-lactams, as well as fosfomycin tromethamine [7]. On the other hand, use of these antibiotics is associated with the development of several side effects including microbial resistance, high cost, low efficacy, a number of life-threatening side effects, as well as repetition of high doses [8]. In this context, several natural approaches have been proposed for the management of UTIs.

Currently, the antimicrobial resistance in *E. coli* is significantly increasing, leading physicians to hesitate in choosing oral antibiotics [4]. In addition, failure to respond to adequate antimicrobial treatment in the first phase of infection requires a further evaluation of disease by radiographic imaging of the urinary tract [9]. Moreover, early severe inflammatory responses to uropathogenic *E. coli* predispose to chronic and recurrent UTIs. In particular, it has been reported that chronic cystitis development is preceded by the release of proinflammatory cytokines 24 h post-infection by *E. coli*, thus inducing bladder mucosal injury. Moreover, patients with chronic cystitis, lasting at least 14 days, are significantly more susceptible to relapses of even more severe chronic cystitis upon bacterial challenge [10]. Further, oxidative stress is implicated in the pathogenesis of cystitis as reactive oxygen species impair bladder function via various pathways [11].

Various natural compounds are known to be effective in preventing and treating acute or chronic UTIs [12].

Bromelain is a complex of proteolytic enzymes obtained from the stem and fruit of the pineapple [*Ananas comosus* (L.) Merr.] through extraction methods. Different preclinical studies have demonstrated that bromelain exerts very important beneficial activities, and it is commonly used as an anti-inflammatory, antimicrobial, and immunomodulatory agent [13,14,15,16]. Moreover, it has been demonstrated that pineapple consumption can reduce the oxidative stress and inflammation in animal models [17,18].

The efficacy and safety of bromelain was confirmed in a number of diseases, such as osteoarthritis, sinusitis [19], as well as pain after periodontal surgery [20], but its role on UTIs has not been clearly defined.

In particular, bromelain exerts antimicrobial effects against various Gram-positive and Gram-negative bacteria, such as *E. coli* [14]. Bromelain was suggested to hinder bacterial growth inducing a protein break in the bacterial cell wall and leading to cell destabilization and lysis [21]. Furthermore, bromelain was also able to prevent bacterial adhesion to specific glycoprotein receptors on the membrane surface [14].

N-acetylcysteine (NAC) is a drug widely used as a mucolytic agent. Its well-known beneficial effects make it extremely useful in multiple diseases [22]. NAC plays a pivotal role in reducing biofilm formation induced by various microorganisms [23,24,25]. In addition, NAC is a potent antioxidant and anti-inflammatory agent since it is able to increase intra-cellular glutathione (GSH) levels and decrease various pro-inflammatory markers, such as tumoral necrosis factor-alpha (TNF-α) and interleukins (IL)-6 and -1β [26,27,28].

Manoharan and collaborators (2010) [29] reported that NAC was able to prevent cell invasion as well as biofilm formation caused by uropathogens of the urinary tract, thus suggesting its potential use in UTIs treatment.

The aim of our study was to evaluate the beneficial effects of a novel exclusive polyenzyme complex with proteolytic activity like bromelain, in association with NAC in UTIs. We evaluated the antibacterial and anti-virulence (anti-motility and anti-biofilm) effects of a proprietary blend of natural ingredients (DIF17BRO^®^) plus NAC (the Formulation) on reference and clinical strains of *E. coli* coming from patients affected by UTIs.

Furthermore, we studied the potential anti-inflammatory and antioxidant activities of the Formulation in an ex vivo model of cystitis, constituted by mouse bladder specimens exposed to *E. coli* lipopolysaccharide (LPS). In particular, we measured cyclooxygenase (COX)-2, nuclear factor-kB (NF-kB), TNF-α, and inducible nitric oxide synthase (iNOS) mRNA levels.

## 2. Results and Discussion

**Figure 1:**
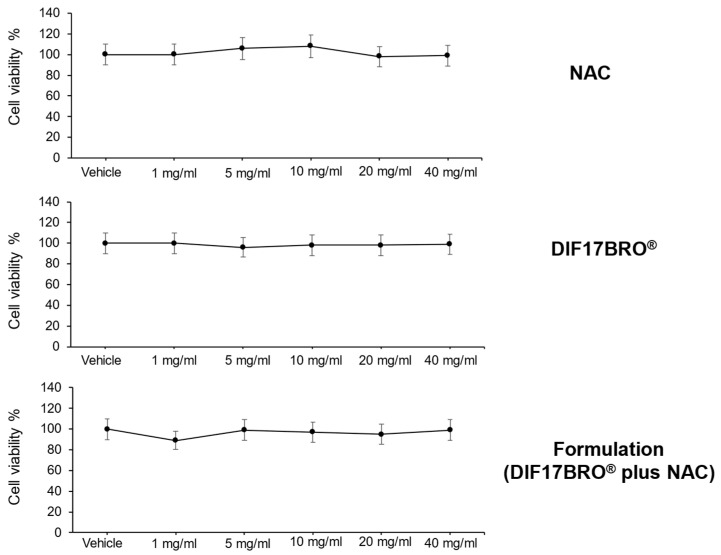
Effects of NAC, DIF17BRO^®^, or Formulation (DIF17BRO^®^ plus NAC) on viability of normal HFF-1 fibroblast cells. Evaluation of cell viability was performed by MTT assay following incubation for 72 h of HFF-1 cells with NAC, DIF17BRO^®^, or Formulation (DIF17BRO^®^ plus NAC) at various concentrations (1-5-10-20-40 mg/mL), or with vehicle (control). Data shown are the means ± SEM of two independent experiments with quintuplicate determinations.

In the first experimental step, we tested NAC, DIF17BRO^®^, and the Formulation (DIF17BRO^®^ plus NAC) at various concentrations (1-5-10-20-40 mg/mL) on the viability of normal HFF-1 fibroblast cells. Our present findings showed that NAC, DIF17BRO^®^, and the Formulation, in the entire evaluated dose range, exhibited no toxicity against normal HFF-1 cells, as shown in the dose response curve (Figure 1).

Our present findings are consistent with previous studies showing that bromelain exerts very low systemic toxicity, in an animal model [24]. In addition, NAC also displays a well-established safety profile, and its toxicity depends on the route of administration and high dosages [17].

In the second experimental step, we evaluated the antibacterial effects of NAC and DIF17BRO^®^ on four strains of *E. coli* (*E. coli* ATCC 10536, *E. coli* ATCC 700926, and clinical isolates *E. coli* PCA, *E. coli* PNT). The minimum inhibitory and bactericidal concentrations of NAC and DIF17BRO^®^ for reference and clinical strains of *E. coli* are reported in Table 1.

As shown in Table 1, NAC showed MIC values of 5 mg/mL for all *E. coli* strains and MBC values of 10 mg/mL except for clinical strain *E. coli* PNT (MBC 5 mg/mL). These results are in accordance with a previous study [30]. Despite bromelain having been described for its antimicrobial activity on Gram-positive and -negative bacteria, in our study no antibacterial effect was recorded for DIF17BRO^®^ (MIC > 40 mg/mL) for each tested strain. A possible limitation of our study is that we have not evaluated the antimicrobial activity of the Formulation.

Therefore, we evaluated the effects of the Formulation on bacterial motility for the same strains of *E. coli*. In this context, it is well known that the bacterial motility plays a crucial role in bacterial survival, host colonization, and/or virulence [31,32].

**Figure 2:**
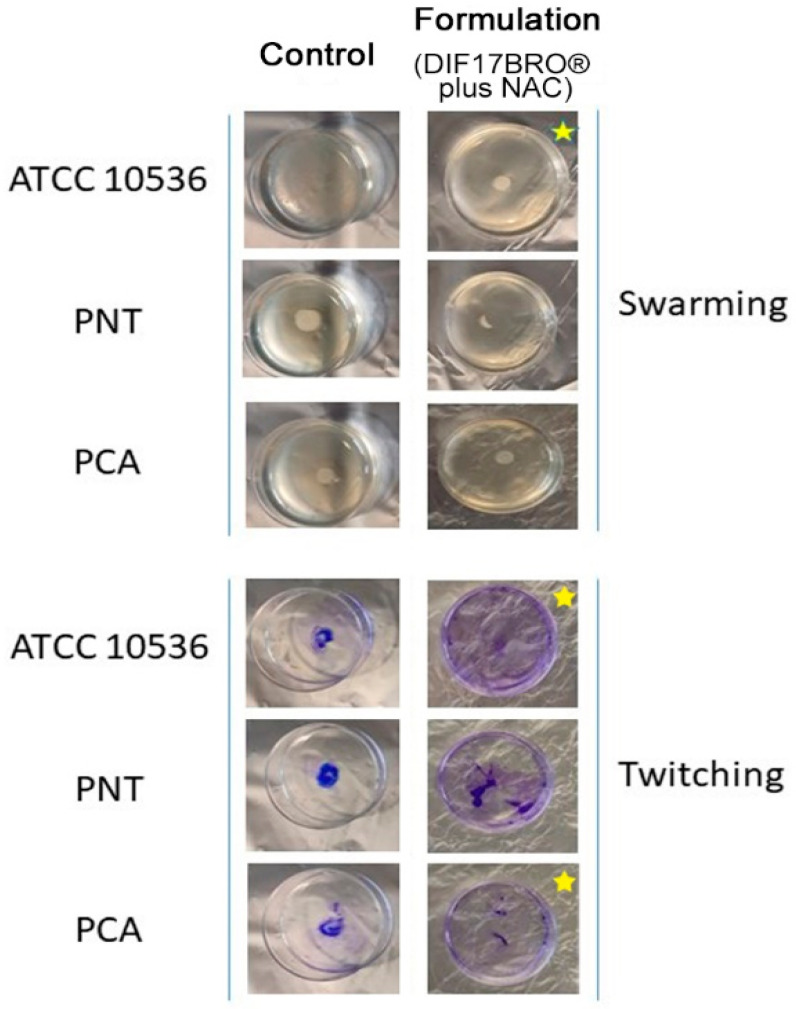
Representative images of macroscopic swarming and twitching assay on cultural plates (3.5 cm diameter Petri dishes) in the presence of Formulation (DIF17BRO^®^ plus NAC) compared to control [untreated sample (medium without Formulation)]. (Halo growth represents bacterial motility). Stars symbol underlines significant values.

The Formulation did not significantly affect the swimming motility in all tested bacteria. Regarding the swarming and twitching motility, the Formulation significantly reduced the halo diameter in *E. coli* ATCC 10536 compared to the control (Figure 2), underlining the capability of the Formulation to affect the swarming behavior unbalancing the coordinated movement of the group of cells. As reported by Rütschlin and Böttcher (2019), swarming motility represents an important dynamic form of coordinated motility regulated by chemical stimuli and by quorum-sensing [33]. Therefore, the Formulation, influencing the swarming phenotype, could contribute to reduce pathogenicity in bacteria. Moreover, the Formulation could affect the expression of flagella, which play a crucial role in the colonization of the upper urinary tract by *E. coli* [33].

In addition to flagellum-mediated motility, uropathogenic bacteria use the type IV pilus-mediated (twitching) motility to promote the spread of bacterial infections to the urinary tract. Twitching motility consists in cycles of pili extension, attachment, and retraction, which represent an important mechanism to colonize the human urinary tissue spreading the infection [34]. From our results, in the twitching motility test, the Formulation decreased the normal adhesive properties of *E. coli* ATCC 10536 and *E. coli* PCA compared to the control (Figure 2), as can be seen from the intensity of crystal violet.

It is well known that the formation of biofilms is an important microbial survival strategy that confers to bacteria the capacity to be up to 1000-fold more tolerant to antibiotics and to evade the action of the host immune system [6]. A number of studies have confirmed the efficacy of NAC in the eradication of biofilm through the cleavage of disulfide bonds which crosslink glycoproteins in biofilm. NAC could prevent cell invasion and intracellular bacterial communities formation by uropathogens, thus providing a potentially novel and efficacious treatment for UTIs [29]. Therefore, we evaluated the antibiofilm activities of the Formulation both on *E. coli* in formation and mature biofilms.

**Figure 3:**
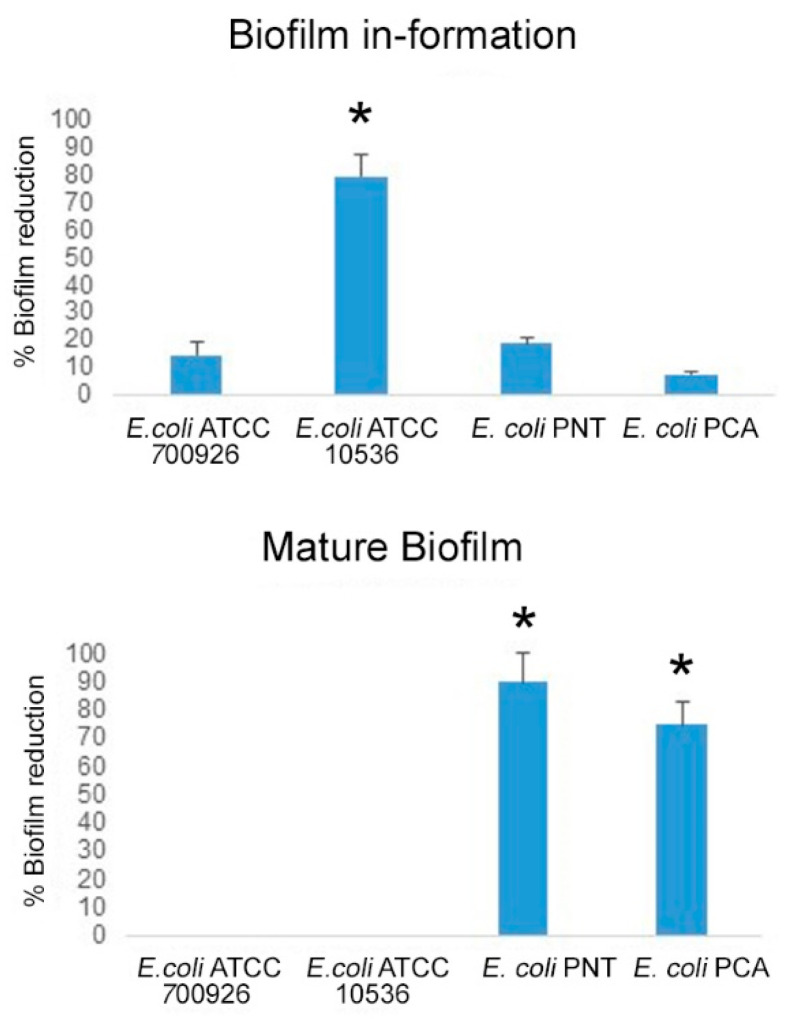
Percentage of reduction in biomass of biofilm in formation and mature biofilm after treatment with Formulation (DIF17BRO^®^ plus NAC). Data are reported as means ± SEM. ANOVA, * *p* < 0.05 vs. reference strains of *E. coli*.

As shown in Figure 3, the Formulation significantly reduced the biomass of biofilm in formation in the *E. coli* ATCC 10536 strain (80.0% reduction). In addition, we demonstrated a reduction in biofilm mature biomass in *E. coli* PNT and PCA clinical strains (89.9% and 74.6% reduction, respectively) after treatment with the Formulation.

**Figure 4:**
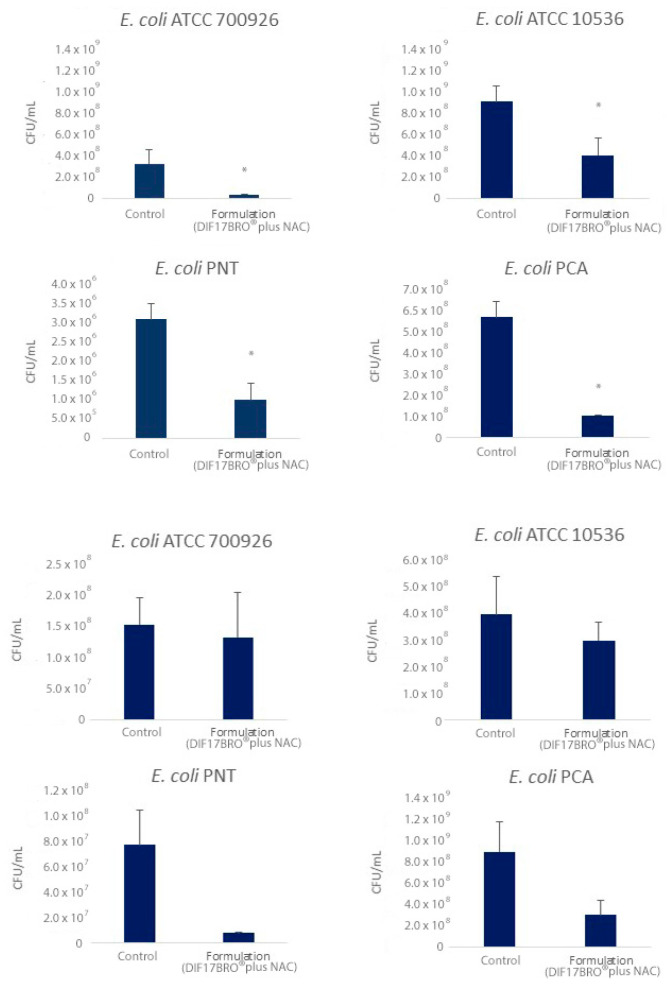
Colony forming unit/mL (CFU/mL) determination of biofilm in formation (up) and mature biofilm (down) for all tested bacteria after treatment with Formulation (DIF17BRO^®^ plus NAC). Control represents the untreated sample. Data are reported as means ± SEM. *t*-test, * *p* < 0.05 vs. control.

Figure 4 shows the effect of the Formulation on CFU/mL of both in-formation and mature biofilm of each tested bacteria. The Formulation significantly affects the capability of bacteria to form biofilm and reduces the bacteria amount in the mature biofilm.

For the biofilm in formation, the Formulation reduced the CFU/mL to 91.1% for *E. coli* ATCC 700926, to 55.6% for *E. coli* ATCC 10536, to 67.7% for *E. coli* PNT, and to 81.8% for *E. coli* PCA. Therefore, it could be hypothesized that a combined effect of the Formulation resulted in the following: inhibition of microbial growth, reduction in bacterial motility, together with an interference with the microbial adhesion. On the other hand, different studies have reported the capacity of bromelain in preventing bacterial adhesion and inhibiting enterotoxin production of *E. coli* [14,31].

**Figure 5:**
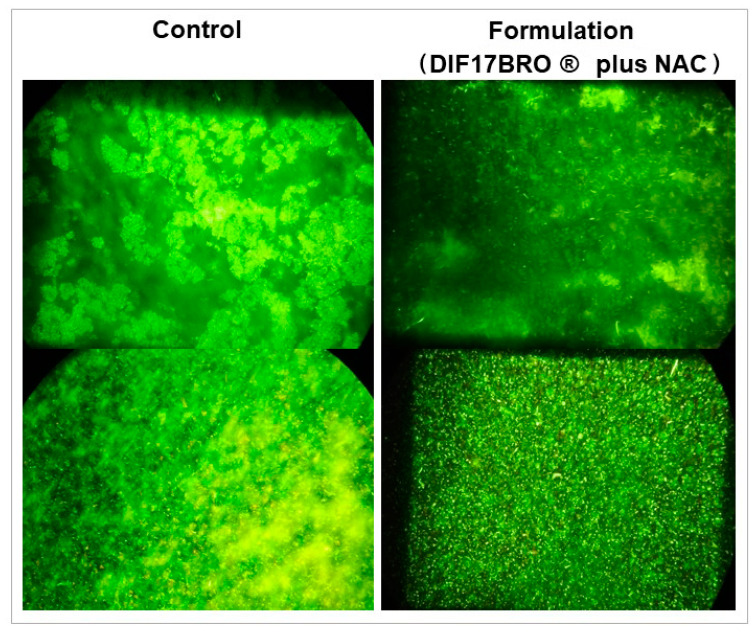
Representative images of *E. coli* ATCC 10536 control and treated with Formulation (DIF17BRO^®^ plus NAC) obtained with live/dead staining (600×, original magnification). Control represents the untreated sample.

For mature biofilm, the Formulation reduced the CFU/mL to 13.3% for *E. coli* ATCC 700926, to 25.0% for *E. coli* ATCC 10536, to 91.0% for *E. coli* PNT, and to 66.7% for *E. coli* PCA. On mature biofilm, the Formulation was capable to significantly reduce the biofilm biomass for all strains in comparison with the control. Moreover, all produced biofilms showed a general detachment from the polystyrene wells, suggesting a possible ability of the Formulation to enter in the polymeric matrix of mature biofilm perturbing and destroying its tridimensional structure. Figure 5 shows representative images of *E. coli* ATCC 10536, the best producer of biofilm, after treatment with the Formulation. It is evident that the mature biofilm exhibits greater disaggregation compared to the structured control after treatment.

As is known, the mechanism of virulence of *E. coli* related to UTIs involves both adhesive structures, such as flagella, pili, adhesins, and capsules, and several toxins, and its capability to adhere and form biofilm is an important virulence factor related to the UTIs [35]. Affecting the *E. coli* virulence, in a multi-target way, could represent an efficacious strategy for the eradicating treatment.

The Formulation studied in this work combines the interesting properties of NAC and a polyenzyme plant complex based on bromelain in a right dose to affect the *E. coli* adhesion capability. NAC shows anti-biofilm activity through the disruption of clinically relevant and drug-resistant bacterial biofilms. Furthermore, since it is capable to improve the activity of antimicrobials in bacteria in biofilm, the Formulation can be considered as an essential enhancer for increasing the antibiotic susceptibility of pathogenic bacteria which can lead to further improved treatment success rates [36].

Bromelain is a potent proteolytic polyenzyme complex that can completely break down a variety of proteins, whose activities can be increased by NAC, as reported by Kumar et al. (2023) [37]. This property could interfere with biofilm formation and facilitate the penetration of antimicrobials through the bacterial biofilm matrix, thus improving the bacterial killing and/or biofilm dispersion actions.

Afterwards, we also evaluated the potential beneficial activities induced by the Formulation in mice bladder specimens stimulated with LPS, which represents a validated model of cystitis [38,39].

COX-2 is highly expressed by neutrophils and urothelial cells in severely infected bladder, and it plays a crucial role in the acute phase of cystitis by pathogens. Hannan and collaborators (2014) suggested that COX-2 inhibition exerts protective activities against severe acute, chronic, and recurrent cystitis by preventing urothelial damage [40]. On the other hand, NF-kB is critically involved in the physiopathogenesis of cystitis; in fact, its activation induces the expression of several proinflammatory factors in the urine of patients with cystitis [41]. In addition, the activation of NF-kB by recombinant human (rh)TNF-α was associated with 27-, eight-, ten-, and seven-fold increases in TNF-α, IL-1β, IL-6, and IL-8 transcripts, respectively [41]. Moreover, various studies reported that patients with cystitis showed a significant increase in reactive oxygen markers levels, which are well known to impair bladder function through multiple molecular mechanisms [42,43].

Therefore, we aimed to investigate the effects of the Formulation on proinflammatory and antioxidative mediators, such as COX-2, NF-kB, TNF-α, and iNOS gene expression on isolated LPS-stimulated bladder specimens, by RT-PCR analysis.

**Figure 6:**
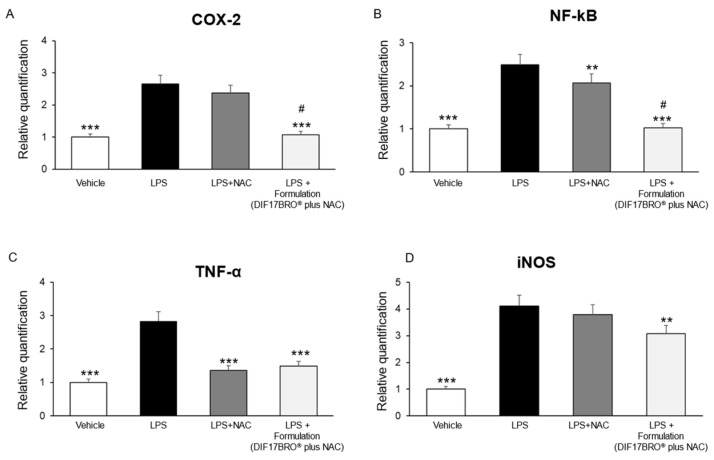
Effects of NAC (5 mg/mL) and Formulation (DIF17BRO^®^ plus NAC; 5 mg/mL + 5 mg/mL) on LPS-induced cyclooxygenase (COX-2) (**A**), nuclear factor-kB (NF-kB) (**B**), tumor necrosis factor (TNF-α) (**C**), and inducible nitric oxide synthase (iNOS) (**D**) gene expression (RQ, relative quantification), in mice bladder specimens. Data are reported as means ± SEM. ANOVA, ** *p* < 0.05 and *** *p* < 0.005 vs. LPS; ^#^ *p* < 0.05 vs. NAC.

In our ex vivo model, we showed that the Formulation was able to reduce COX-2, NF-kB, TNF-α, and iNOS gene expression (Figure 6).

Although the mechanisms for bromelain’s anti-inflammatory activity have not been well understood, bromelain has been suggested to exert beneficial effects on human health by modulating multiple inflammatory mediators [44]. In agreement, preclinical studies showed that bromelain is able to downregulate different proinflammatory mediators, such as NF-kB, COX-2, and TNF-α [45,46,47].

In addition, as shown in Figure 6, the Formulation markedly reduced the gene expression of COX-2 and NF-kB compared to NAC alone.

Therefore, we accomplished a new formulation that was able not only to inhibit pathogen adhesion, thus contrasting with biofilm formation, but also to exert an antioxidant and anti-inflammatory direct function on the bladder. Similarly, Akhter and collaborators (2021) have demonstrated that the combination of bromelain’s multipotent enzymatic properties with acetylcysteine leads to a synergic action [48].

## 3. Materials and Methods

### 3.1. Characteristics of Blend of Natural Ingredients DIF17BRO^®^

DIF17BRO^®^ (Lot N°: B01/A9/23) and NAC (Lot N°: HADD23030602) were supplied as dried powder by Difass International S.p.a. (Coriano, Rimini, Italy). DIF17BRO^®^ is an exclusive polyenzyme complex with proteolytic activity, based on bromelain (Patent n° 102016000109433). As reported in Table 2, it is a blend of fruit and stem extracts of *Ananas comosus* (L.) Merr. with enzymatic activity of 1.825 GDU/g.

DIF17BRO^®^ was dissolved in sterile phosphate buffer solution (PBS) and NAC was dissolved in 20% DMSO to prepare the stock solution (100 mg/mL). Then, for the experimental evaluations, the stock solution was dissolved in sterile PBS.

### 3.2. Cell Lines and Treatments

Human fibroblast cell line HFF-1 was purchased from American Type Culture Collection (ATCC; Manassas, VA, USA) and it was cultured in DMEM high glucose (4.5 g/L), supplemented with 15% FBS, 1% Pen/Strep, and 1% L-glutamine. The cell line was maintained in a humidified incubator at 37 °C, 5% CO_2_.

### 3.3. Cell Viability Assay

Cell viability was evaluated by the MTT assay [3-(4,5-Dimethyl-2-thiazolyl)-2,5-diphenyl-2H-tetrazolium bromide] (Sigma, St. Louis, MO, USA), as previously described [49]. Briefly, the HFF-1 cell line was seeded in 96-well plates (5 × 10^3^ cells/well) and was treated the following day with DIF17BRO^®^, NAC, and the Formulation (DIF17BRO^®^ plus NAC) at various concentrations (1-5-10-20-40 mg/mL), or with the vehicle (control). After 72 h of treatment, the MTT solution was added to each well and incubated at 37 °C for at least 3 h, until purple formazan crystals were formed. The detailed protocol is described in the Supplementary Materials Section.

### 3.4. Microbial Cultures

Reference strains *E. coli* ATCC 10536 and *E. coli* ATCC 700926, and clinical isolates *E. coli* PCA and *E. coli* PNT, coming from the private collection of Bacteriological Laboratories of Dept. Pharmacy of University “G. d’Annunzio” Chieti-Pescara, were used in this study.

Bacteria were cultured on MacConkey agar (MK, Oxoid, Milan, Italy); for the experiments, fresh pure bacterial colonies were cultured in Trypticase Soy Broth (TSB, Oxoid, Milan, Italy) and incubated at 37 °C overnight in aerobic condition.

### 3.5. Antimicrobial Activity

The antibacterial effects of DIF17BRO^®^, NAC, and the Formulation (DIF17BRO^®^ plus NAC) were evaluated by minimum inhibitory concentration and minimum bactericidal concentration (MIC, MBC) determination following the broth microdilution method according to EUCAST guidelines [50]. The detailed protocol is described in the Supplementary Materials Section. Bacteria in MHB without DIF17BRO^®^/NAC and sterile MHB were used as positive and negative controls, respectively. MIC was defined as the lowest concentration of substances that induces a complete growth inhibition. MBC was defined as the lowest concentration at which no bacterial growth occurred on Mueller–Hinton agar plates.

All experiments were performed for three independent experiments, in duplicate.

### 3.6. Evaluation of the Formulation (DIF17BRO^®^ Plus NAC)’s Activity on Bacterial Anti-Swim/Swarm and Anti-Twitch

The Formulation’s capability to interfere with the swarming and swimming motility was evaluated according with the previously reported methodologies [32] (Supplementary Materials Section). For the swarming motility, each standardized culture was inoculated at the center of the swarming plates medium containing 1% peptone, 0.5% NaCl, 0.5% agar, and 0.5% D-glucose in the presence of each substance (at sub-inhibitory concentration, ¼ MIC) or without the Formulation (control). For the swimming motility, the cultures were inoculated at the center of the plates medium containing 1% peptone, 0.5% NaCl, 0.5% agar, and 0.5% D-glucose in the presence of each substance (at sub-inhibitory concentration, ¼ MIC) or without the Formulation (control). For twitching motility, each standardized bacterial culture was inoculated to the bottom of the twitching plates containing 10 g/L of tryptone, 5 g/L of yeast extract, 10 g/L of NaCl, and 1% agar with each substance (at sub-inhibitory concentration, ¼ MIC) or without the Formulation (control).

To evaluate the anti-biofilm formation effect of the Formulation against *E. coli* reference and clinical strains, bacterial suspensions were standardized as described above, and incubated with the Formulation (DIF17BRO^®^ plus NAC; 5 mg/mL + 5 mg/mL) or without the Formulation (control, untreated sample) in TSB supplemented with 1% glucose in flat-bottomed microtiter 96-wells polystyrene plates and incubated for 24 h at 37 °C, in aerobic condition.

### 3.7. Anti-Biofilm Effect of the Formulation (DIF17BRO^®^ Plus NAC)

To evaluate the effect of the Formulation on mature biofilm produced by *E. coli* reference and clinical strains, the standardized microbial suspensions, grown in TSB supplemented with 1% glucose, were incubated on 96-well flat-bottomed polystyrene microtiter plates under aerobic condition for 24 h. After incubation, the supernatant was discharged and the adherent mature biofilms were washed and treated with the Formulation (DIF17BRO^®^ plus NAC; 5 mg/mL + 5 mg/mL) for 24 h. Bacteria in TSB supplemented with 1% glucose without DIF17BRO^®^/NAC and sterile TSB supplemented with 1% glucose were used as positive and negative controls, respectively.

The detailed protocol is described in the Supplementary Materials Section.

The mature treated and untreated biofilms were observed under a fluorescent microscope after staining with the live/dead kit [51].

All evaluations were performed for three independent experiments.

### 3.8. Animals

Adult C57/BL6 male mice (3-month-old, weight 20–25 g) (*n* = 12) were housed in plexiglass cages (2–4 animals per cage; 55 × 33 × 19 cm) and maintained as reported in the Supplementary Materials Section. Housing conditions and experimentation procedures were strictly in agreement with the European Community ethical regulations (EU Directive no. 63/2010) on the care of animals for scientific research. According to the recognized principles of “Replacement, Refinement, and Reduction in Animals in Research”, bladder specimens were obtained as residual material from vehicle-treated animals randomized in our previous experiments, approved by the local ethical committee (‘G. d’Annunzio’ University, Chieti, Italy) and Italian Health Ministry (Project no. 885/2018-PR).

### 3.9. Ex Vivo Studies

After collection, isolated bladder specimens (*n* = 12) were maintained in a humidified incubator with 5% CO_2_ at 37 °C for 4 h, in RPMI buffer with added bacterial LPS (Sigma-Aldrich, St. Louis, MO, USA) (10 μg/mL) (incubation period), as previously reported [39,52]. During the incubation period, bladder specimens were treated with NAC (5 mg/mL) and the Formulation (DIF17BRO^®^ plus NAC; 5 mg/mL + 5 mg/mL). Total RNA was extracted from the bladder specimens using TRI Reagent (Sigma-Aldrich, St. Louis, MO, USA), according to the manufacturer’s protocol. Gene expression of COX-2, TNF-α, NF-kB, and iNOS was measured by quantitative real-time PCR using TaqMan probe-based chemistry, as previously described [53,54] (Supplementary Materials Section).

### 3.10. Statistical Analysis

The data were analyzed by the licensed software GraphPad Prism version 6.0 (Graph-pad Software Inc., San Diego, CA, USA). Analysis of means ± SEM for each experimental group was performed by *t*-test and one-way analysis of variance (ANOVA), followed by either the Newman–Keuls multiple comparisons post hoc test or by the Bonferroni post hoc test.

## 4. Conclusions

In conclusion, our results showed that our blend of natural ingredients (DIF17BRO^®^) in association with NAC exhibited protective effects, as confirmed by the inhibitory activities on multiple inflammatory and oxidative stress-related pathways on bladder specimens exposed to LPS.

Moreover, the Formulation significantly affects the capability of bacteria to form biofilm and reduces the bacteria amount in the mature biofilm. Moreover, it combines the interesting properties of NAC and a polyenzyme plant complex based on bromelain in a right dose to affect the *E. coli* adhesion capability.

This blend of active compounds could represent a promising and versatile approach to use in the treatment of UTIs due to *E. coli* to overcome the limitations associated with conventional therapies, because it could represent an efficacious and cost-effective strategy.

However, further studies are necessary to confirm the effectiveness of the Formulation as well as to further deepen knowledge of the mechanisms involved in its activities. Moreover, more research is required to evaluate potential synergic effects of the Formulation in combination with antibiotic therapy.

## Figures and Tables

**Table 1:** Minimum inhibitory concentration (MIC) and minimum bactericidal concentration (MBC) of NAC and DIF17BRO^®^ for reference and clinical strains of *E. coli*.

Bacteria	MIC	MBC
*E. coli* ATCC 10536	NAC: 5 mg/mL DIF17BRO^®^: >40 mg/mL	NAC: 10 mg/mL DIF17BRO^®^: >40 mg/mL
*E. coli* ATCC 700926	NAC: 5 mg/mL DIF17BRO^®^: >40 mg/mL	NAC: 10 mg/mL DIF17BRO^®^: >40 mg/mL
*E. coli* PNT	NAC: 5 mg/mL DIF17BRO^®^: >40 mg/mL	NAC: 5 mg/mL DIF17BRO^®^: >40 mg/mL
*E. coli* PCA	NAC: 5 mg/mL DIF17BRO^®^: >40 mg/mL	NAC: 10 mg/mL DIF17BRO^®^: >40 mg/mL

**Table 2:** Characteristics of blend of natural ingredients DIF17BRO^®^.

Botanical family	Bromeliaceae
Botanical name	*Ananas comosus* (L.) Merr.
Part of plant used	Fruit and stem
Appearance	Off-white/beige fine powder
Proteolytic activity as bromelain	1.825 GDU/g *
Loss on drying	<=5%

* Potency of bromelain compounds is quantified in GDUs (gelatin dissolving units).

## Data Availability

The data that support the findings of this study are available from the corresponding authors upon reasonable request.

## Supplementary Materials

The following supporting information can be downloaded at: https://www.mdpi.com/article/10.3390/antibiotics13100985/s1.
