# Supplementary material for: Beneficial Effects Induced by a Proprietary Blend of a New Bromelain-Based Polyenzymatic Complex Plus N-Acetylcysteine in Urinary Tract Infections: Results from In Vitro and Ex Vivo Studies
Source: Antibiotics (Basel). 2024 Oct 18;13(10):985. doi: 10.3390/antibiotics13100985 (PMC11505172; doi:10.3390/antibiotics13100985)
Supplement: Supplementary file 1 [file antibiotics-13-00985-s001.zip › antibiotics-3240183-supplementary.pdf]

antibiotics

MDPI

Article

Beneficial Effects Induced by a Proprietary Blend of a New Bromelain-Based Polyenzymatic Complex Plus N-Acetylcysteine in Urinary Tract Infections: Results from In Vitro and Ex Vivo Studies

Lucia Recinella [1,†], Morena Pinti [1,†], Maria Loreta Libero [1,*], Silvia Di Lodovico [1], Serena Veschi [1], Anna Piro [1], Daniele Generali [2,3], Alessandra Acquaviva [1], Nilofar Nilofar [1], Giustino Orlando [1], Annalisa Chiavaroli [1], Claudio Ferrante [1], Luigi Menghini [1], Simonetta Cristina Di Simone [1], Luigi Brunetti [1], Mara Di Giulio [1] and Sheila Leone [1]

1 Department of Pharmacy, "G. d'Annunzio" University, 66013 Chieti, Italy; lucia.recinella@unich.it (L.R.); morena.pinti@phd.unich.it (M.P.); silvia.dilodovico@unich.it (S.D.L.); veschi@unich.it (S.V.); anna.piro@unich.it (A.P.); alessandra.acquaviva@unich.it (A.A.); nilofar.nilofar@unich.it (N.N.); giustino.orlando@unich.it (G.O.); annalisa.chiavaroli@unich.it (A.C.); claudio.ferrante@unich.it (C.F.); luigi.menghini@unich.it (L.M.); simonetta.disimone@unich.it (S.C.D.S.); luigi.brunetti@unich.it (L.B.); mdigiulio@unich.it (M.D.G.); sheila.leone@unich.it (S.L.)

2 Department of Medical, Surgical and Health Sciences, University of Trieste, 34149 Trieste, Italy; dgenerali@units.it

3 Department of Advanced Translational Microbiology, Institute for Maternal and Child Health-IRCCS "Burlo Garofolo", 34137 Trieste, Italy

* Correspondence: maria.libero@unich.it; Tel.: +39-0871-3554673

† These authors contributed equally to this work.

Abstract: Background/Objectives: Urinary tract infections (UTIs) are infections that involve the urethra, bladder, and, in much more severe cases, even kidneys. These infections represent one of the most common diseases worldwide. Various pathogens are responsible for this condition, the most common being *Escherichia coli* (*E. coli*). Bromelain is a proteolytic complex obtained from the stem and stalk of *Ananas comosus* (L.) Merr. showing several beneficial activities. In addition to bromelain, N-acetylcysteine (NAC) has also been used. **Methods:** The purpose of this experiment was to evaluate the antibacterial, anti-motility, and anti-biofilm effects of a new polyenzymatic complex (DIF17BRO®) in combination with NAC (the Formulation) on various strains of *E. coli* isolated from patients with UTIs. Subsequently, the anti-inflammatory and antioxidant effects of the Formulation were studied in an ex vivo model of cystitis, using bladder samples from mice exposed to *E. coli* lipopolysaccharide (LPS). **Results:** Our results showed that the Formulation significantly affects the capability of bacteria to form biofilm and reduces the bacteria amount in the mature biofilm. Moreover, it combines the interesting properties of NAC and a polyenzyme plant complex based on bromelain in a right dose to affect the *E. coli* adhesion capability. Finally, the Formulation exhibited protective effects, as confirmed by the inhibitory activities on multiple inflammatory and oxidative stress-related pathways on bladder specimens exposed to LPS. **Conclusions:** This blend of active compounds could represent a promising and versatile approach to use to overcome the limitations associated with conventional therapies.

Keywords: bromelain; N-acetylcysteine; adhesion capability; biofilm; oxidative stress; inflammation

S1. Materials and Methods

S1.1. Characteristics of Blend of Natural Ingredients DIF17BRO®

DIF17BRO® (Lot N°: B01/A9/23) and NAC (Lot N°: HADD23030602) were supplied as dried powder by Difass International S.p.a. (Coriano, Rimini, Italy). DIF17BRO® is an exclusive polyenzyme complex with proteolytic activity, based on bromelain (Patent n° 102016000109433). As reported in Table 2 (in the main text), it is a blend of fruit and stem extracts of *Ananas comosus* (L.) Merr. with enzymatic activity of 1.825 GDU/g.

DIF17BRO® was dissolved in sterile phosphate buffer solution (PBS) and NAC was dissolved in 20% DMSO to prepare the stock solution (100 mg/mL). Then, for the experimental evaluations, the stock solution was dissolved in sterile PBS.

S1.2. Cell Lines and Treatments

Human fibroblast cell line HFF-1 was purchased from American Type Culture Collection (ATCC; Manassas, VA, USA) and it was cultured in DMEM high glucose (4.5 g/L), supplemented with 15% FBS, 1% Pen/Strep and 1% L-glutamine. Cell line was maintained in a humidified incubator at 37°C, 5% CO_2.

S1.3. Cell Viability Assay

Cell viability was evaluated by MTT assay [3-(4,5-Dimethyl-2-thiazolyl)-2,5-diphenyl-2H-tetrazolium bromide] (Sigma, St. Louis, MO, USA), as previously described [1]. Briefly, HFF-1 cell line was seeded in 96-well plates ($5x10^3$ cells/well) and was treated the following day with DIF17BRO®, NAC and Formulation (DIF17BRO® plus NAC) at various concentrations (1-5-10-20-40 mg/ml), or with vehicle (control). After 72 hours of treatment, the MTT solution was added to each well and incubated at 37°C for at least 3 hours, until purple formazan crystals were formed. In order to dissolve the precipitate, the culture medium was replaced with dimethyl sulfoxide (DMSO, Euroclone). Absorbance of each well was quantified at 540 and 690 nm, using a Synergy H1 microplate reader (BioTek Instruments Inc., Winooski, VT, USA). IC50 values were calculated using the CompuSyn software.

S1.4. Microbial Cultures

Reference strains *E. coli* ATCC 10536, *E. coli* ATCC 700926 and clinical isolates *E. coli* PCA, *E. coli* PNT coming from the private collection of Bacteriological Laboratories of Dept. Pharmacy of University "G. d'Annunzio" Chieti-Pescara, were used in this study.

Bacteria were cultured on MacConkey agar (MK, Oxoid, Milan, Italy); for the experiments, fresh pure bacterial colonies were cultured in Trypticase Soy Broth (TSB, Oxoid) and incubated at 37 °C overnight in aerobic condition.

S1.5. Antimicrobial Activity

The antibacterial effects of DIF17BRO®, NAC and Formulation (DIF17BRO® plus NAC) were evaluated by Minimum Inhibitory and Minimum Bactericidal Concentrations (MIC, MBC) determination following the broth-microdilution-method according to EUCAST guidelines [2]. From overnight cultures, bacteria were refreshed in Mueller–Hinton Broth (MHB) for 2 h at 37 °C in an orbital shaker in aerobic condition and standardized at ~ 5×10^7 CFU/mL. Then, each strain was incubated on microtiter plates at several concentrations of NAC and DIF17BRO® (0.07-40 mg/mL) for 24 h at 37°C. Bacteria in MHB without DIF17BRO®/ NAC and sterile MHB were used as positive and negative controls, respectively. MIC was defined as the lowest concentration of substances that induces a complete growth inhibition. MBC was defined as the lowest concentration at which no bacterial growth occurred on Mueller–Hinton Agar plates.

All experiments were performed for three independent experiments, in duplicate.

S1.6. Evaluation of the Formulation (DIF17BRO® Plus NAC) Activity on Bacterial Anti-Swim/Swarm and Anti-Twitch

Formulation capability to interfere with the swarming and swimming motility was evaluated according with the previously reported methodologies [3]. Briefly, for the swarming motility, each standardized culture was inoculated at the center of swarming plates medium containing 1% peptone, 0.5% NaCl, 0.5% agar, and 0.5% D-glucose in presence of each substance (at sub-inhibitory concentration, ¼ MIC) or without Formulation (Control); for twitching motility, and 1% agar) For the swimming motility, the cultures were inoculated at the center of plates medium containing 1% peptone, 0.5% NaCl, 0.5% agar, and 0.5% D-glucose 0.3% agar in presence of each substance (at sub-inhibitory concentration, ¼ MIC) or without Formulation (Control). After incubation, bacterial halos were measured and compared to the controls. For twitching motility, each standardized bacterial cultures was inoculated to the bottom of the twitching plates containing 10 g/L of tryptone, 5 g/L of yeast extract, 10 g/L of NaCl and 1% agar with each substance (at sub-inhibitory concentration, ¼ MIC) or without Formulation (Control). After incubation, the agar was removed, and the halos were stained with 0.1% Crystal Violet, measured and compared to the controls.

To evaluate the anti-biofilm formation effect of the Formulation against *E. coli* reference and clinical strains, bacterial suspensions were standardized as described above, and incubated with Formulation (DIF17BRO® plus NAC; 5mg/ml + 5mg/ml) or without Formulation (Control, untreated sample) in TSB supplemented with 1% glucose in flat bottomed microtiter 96-wells of polystyrene plates and incubated for 24 h at 37°C, in aerobic condition.

S1.7. Anti-Biofilm Effect of the Formulation (DIF17BRO® Plus NAC)

To evaluate the effect of the Formulation on mature biofilm produced by *E. coli* reference and clinical strains, the standardized microbial suspensions, grown in TSB supplemented with 1% glucose were incubated on 96-wells flat-bottomed polystyrene microtiter plate under aerobic condition for 24 h. After incubation, the supernatant was discharged and the adherent mature biofilms were washed and treated with Formulation (DIF17BRO® plus NAC; 5mg/ml + 5mg/ml) for 24 h. Bacteria in TSB supplemented with 1% glucose without DIF17BRO®/ NAC and sterile TSB supplemented with 1% glucose were used as positive and negative controls, respectively.

After incubation, for both biofilm formation and mature biofilm, the supernatants were removed, and the determination of CFU/mL of biofilm was carried out by scraping, vortexing and dilution and spreading on MK. For the biomass quantification, the biofilms were dried, stained for 1 min with 0.1% Safranin, and eluted with ethanol. The biomasses were measured by spectrophotometer using an enzyme-linked-immunoadsorbent assay (ELISA) reader OD_{492} nm. The mature treated and untreated biofilm were observed under fluorescent microscope after staining with Live/dead kit [4]. All evaluations were performed for three independent experiments.

S1.8. Animals

Adult C57/BL6 male mice (3-month-old, weight 20–25 g) (n=12) were housed in Plexiglass cages (2–4 animals per cage; 55 × 33 × 19 cm) and maintained under standard laboratory conditions (21 ± 2 °C; 55 ± 5% humidity) on a 14/10 h light/dark cycle, with ad libitum access to water and normal laboratory chow (RMH-B diet, Arie Blok animal feed, Worden, the Netherlands). Housing conditions and experimentation procedures were strictly in agreement with the European Community ethical regulations (EU Directive no. 63/2010) on the care of animals for scientific research. According to the recognized principles of "Replacement, Refinement and Reduction in Animals in Research", bladder specimens were obtained as residual material from vehicle-treated animals randomized in our

previous experiments, approved by the local ethical committee ('G. d'Annunzio' University, Chieti, Italy) and Italian Health Ministry (Project no. 885/2018-PR).

S1.9. Ex Vivo Studies

Mice were sacrificed by CO_2 inhalation (100% CO_2 at a flow rate of 20% of the chamber volume per min), and bladder specimens (n=12) were immediately collected and maintained in a humidified incubator with 5% CO_2 at 37 °C for 4 h, in RPMI buffer with added bacterial LPS (Sigma–Aldrich, St. Louis, MO, USA) (10 μg/mL) (incubation period) [5,6]. During the incubation period, bladder specimens were treated with NAC (5mg/ml) and Formulation (DIF17BRO® plus NAC; 5mg/ml + 5mg/ml). After collection, total RNA was extracted from the colon specimens using TRI Reagent (Sigma–Aldrich, St. Louis, MO, USA), according to the manufacturer's protocol. Contaminating DNA was removed using 2 units of RNase-free DNase 1 (DNA-free kit, Ambion, Austin, TX, USA). The RNA concentration was quantified at 260 nm by spectrophotometer reading (BioPhotometer, Eppendorf, Hamburg, Germany) and its purity was assessed by the ratio at 260 and 280 nm readings. The quality of the extracted RNA samples was also determined by electrophoresis through agarose gels and staining with ethidium bromide, under UV light. One microgram of total RNA extracted from each sample in a 20 μL reaction volume was reverse transcribed using High Capacity cDNA Reverse Transcription Kit (Thermo Fisher Scientific Inc., Monza, Italy). Reactions were incubated in a 2720 Thermal Cycler (Thermo Fisher Scientific Inc., Monza, Italy) initially at 25 °C for 10 min, then at 37 °C for 120 min, and finally at 85 °C for 5 s. Gene expression of COX-2, TNF-α, NF-kB, and iNOS was determined by quantitative real-time PCR using TaqMan probe-based chemistry, as previously described [7]. β-actin (Thermo Fisher Scientific Inc., Monza, Italy, Part No. 4352340E) was used as the housekeeping gene. The real-time PCR was carried out in triplicate for each cDNA sample in relation to each of the investigated genes. Data were elaborated with the Sequence Detection System (SDS) software version 2.3 (Thermo Fisher Scientific Inc., Monza, Italy). Gene expression was relatively quantified by the comparative $2^{-\Delta\Delta Ct}$ method [8].

S1.10. Statistical Analysis

The data were analyzed by the licensed software GraphPad Prism version 6.0 (Graph-pad Software Inc., San Diego, CA, USA). Analysis of means ± SEM for each experimental group was performed by t-test and one-way analysis of variance (ANOVA), followed by either the Newman-Keuls multiple comparison post hoc test or by the Bonferroni post hoc test.

References

1. Veschi, S.; De Lellis, L.; Florio, R.; Lanuti, P.; Massucci, A.; Tinari, N.; De Tursi, M.; di Sebastiano, P.; Marchisio, M.; Natoli, C.; et al. Effects of Repurposed Drug Candidates Nitroxoline and Nelfinavir as Single Agents or in Combination with Erlotinib in Pancreatic Cancer Cells. *J Exp Clin Cancer Res* **2018**, 37, 236, doi:10.1186/s13046-018-0904-2.
2. Arendrup, M.C.; Cuenca-Estrella, M.; Lass-Flörl, C.; Hope, W.; EUCAST-AFST EUCAST Technical Note on the EUCAST Definitive Document EDef 7.2: Method for the Determination of Broth Dilution Minimum Inhibitory Concentrations of Antifungal Agents for Yeasts EDef 7.2 (EUCAST-AFST). *Clin Microbiol Infect* **2012**, 18, E246-247, doi:10.1111/j.1469-0691.2012.03880.x.
3. Palma, V.; Gutiérrez, M.S.; Vargas, O.; Parthasarathy, R.; Navarrete, P. Methods to Evaluate Bacterial Motility and Its Role in Bacterial-Host Interactions. *Microorganisms* **2022**, 10, 563, doi:10.3390/microorganisms10030563.
4. Di Giulio, M.; Zappacosta, R.; Di Lodovico, S.; Di Campli, E.; Siani, G.; Fontana, A.; Cellini, L. Antimicrobial and Antibiofilm Efficacy of Graphene Oxide against Chronic Wound Microorganisms. *Antimicrob Agents Chemother* **2018,** 62, e00547-18, doi:10.1128/AAC.00547-18.
5. Recinella, L.; Gorica, E.; Chiavaroli, A.; Fraschetti, C.; Filippi, A.; Cesa, S.; Cairone, F.; Martelli, A.; Calderone, V.; Veschi, S.; et al. Anti-Inflammatory and Antioxidant Effects Induced by Allium Sativum L. Extracts on an Ex Vivo Experimental Model of Ulcerative Colitis. *Foods* **2022**, 11, 3559, doi:10.3390/foods11223559.
6. Recinella, L.; Libero, M.L.; Citi, V.; Chiavaroli, A.; Martelli, A.; Foligni, R.; Mannozzi, C.; Acquaviva, A.; Di Simone, S.; Calderone, V.; et al. Anti-Inflammatory and Vasorelaxant Effects Induced by an Aqueous Aged Black Garlic Extract Supplemented with Vitamins D, C, and B12 on Cardiovascular System. *Foods* **2023**, 12, 1558, doi:10.3390/foods12071558.

7. Libero, M.L.; Lucarini, E.; Recinella, L.; Ciampi, C.; Veschi, S.; Piro, A.; Chiavaroli, A.; Acquaviva, A.; Nilofar, N.; Orlando, G.; et al. Anti-Inflammatory and Anti-Hyperalgesic Effects Induced by an Aqueous Aged Black Garlic Extract in Rodent Models of Ulcerative Colitis and Colitis-Associated Visceral Pain. *Phytother Res* **2024**, 38, 4177–4188, doi:10.1002/ptr.8270.
8. Livak, K.J.; Schmittgen, T.D. Analysis of Relative Gene Expression Data Using Real-Time Quantitative PCR and the 2(-Delta Delta C(T)) Method. *Methods* **2001**, 25, 402–408, doi:10.1006/meth.2001.1262.

Disclaimer/Publisher's Note: The statements, opinions and data contained in all publications are solely those of the individual author(s) and contributor(s) and not of MDPI and/or the editor(s). MDPI and/or the editor(s) disclaim responsibility for any injury to people or property resulting from any ideas, methods, instructions or products referred to in the content.
